# Sanatoria for Consumptives

**Published:** 1905-12-02

**Authors:** 


### The Hospital.

A JOURNAL OF

The Medical Sciences and Hospital Administration.

VOL. XXXIX.—No. 1,001. SATURDAY, DECEMBER 2, 1905.

### SANATORIA FOR CONSUMPTIVES.

THE efforts that are now being made in various directions to establish sanatoria for consumptives must, as far as their immediate objects and tendencies are concerned, command the sympathy both of sanitary reformers and of the classes somewhat vaguely described as the benevolent. Sir William Broadbent, in a recent letter to the *Times*, has given terse expression to notorious facts in saying that "the infectious character of tuberculosis is recognised, the way in which it is communicated from person to person is known, the methods by which its spread may be prevented have been pointed out; but up to the present time little has been done by the public authorities, which alone have it in their power to carry out the measures required." We must confess to a considerable doubt whether "public authorities" have indeed the power which Sir William ascribes to them; and we feel sure that any effective attempt on their part to restrain the spread of tuberculosis would be met by some new development of resistance to sanitation. The only possible foundation for such restraint would be compulsory notification, and public opinion is not sufficiently educated to support any action by which persons in an early stage of phthisis could be prevented from scattering abroad their bacilli. An "Anti-prevention of Spitting Society" would be formed, and would command the sympathy and support not only of certain mischievous societies now existing, but also of the fools, if such there were, whom the societies in question had left untouched. Some considerable provincial town would perceive an opportunity for becoming famous, and would take consumption to its bosom, as Leicester and Derby have taken small-pox. Municipal and even Parliamentary elections would turn upon the preservation of the inherent right of man to expectorate, and the leaders of political parties would cause it to be known that their minds were open upon the question, and that they were unfavourable to a "tyranny of doctors." The only hope of the continuance of the recent diminution of tuberculosis must rest, we think, upon the continued and gradual operation of the influences by which that diminution has been brought about in the past, and upon the success which may attend upon educational efforts in the future. In the conduct of the latter sanatoria seem likely to occupy a prominent place. The patients who are received into them are taught, by personal experience as well as by precept, the benefits which attend upon free exposure to air and sunlight, and they are guided into the adoption of precautions which are calculated to check the diffusion of their disease. Many, of course, with the recklessness of selfish ignorance, will revert to their old habits as soon as they return to their homes and occupations, and, as a natural consequence, will speedily lose any benefit which they may have received, and will continue to expose their families to danger. Still, on the whole, there can be no doubt that much good may in this way be effected; and, if the question of sanatoria were taken up, as there seems some prospect that it will be, by the authorities of the great benefit societies, the results would quite certainly receive expression in figures which it would be impossible for the ruling bodies of these societies to ignore. They would be compelled, at no distant time, to protect themselves by their rules against the losses which neglected phthisis now entails upon them—losses the avoidable character of which would be fully established by experience.

In order, however, that such desirable results may be brought about, it seems to us essential that the ambitions of the architect and of the builder should be restrained, and that the sanatoria should consist, in the main, of buildings of a cheap and even of a temporary character. Prior to the discoveries of Lord Lister, and at a time when the increase in the number of surgical operations due to the use of anæsthetics was attended by an appalling mortality, it was gravely proposed that the ordinary hospitals of the future should be only temporary buildings, which might be burnt down as soon as they became dangerously saturated with emanations from the sick; and no doubt was entertained that structures of galvanised iron and boarding could be made to fulfil every hospital requirement. They could certainly be made to fulfil all the requirements of sanatoria.

If sanatoria are to be made really effective engines for dealing with a great national evil, they must be so numerous that fitting cases will have no difficulty in gaining admission to them; and they must be open, not merely to the early stages of pulmonary phthisis, but also to the many other forms of tuberculosis, probably, on the whole, equal to those of pulmonary phthisis in number, by which chronic disablement and premature death are so constantly produced. Cheapness of buildings is a condition absolutely essential to the attainment of an amount of accommodation sufficient to make any real impression upon the extension of the disease, and we may well be content with buildings of a temporary character in the hope that the needs which they are designed to meet will themselves be temporary also. If there be any future in medicine, we may reasonably expect the cure of tuberculosis by some form of antitoxin, and the consequent disappearance of the now formidable character of the disease. The cases treated by T R tuberculin by Dr. A. E. Wright, and exhibited by him last year at St. Mary's Hospital to the French physicians and surgeons visiting London, are alone full of promise, as also is the work now being conducted by Dr. Behring, of Marburg, to whom, in conjunction with Dr. Roux, we are already indebted for the antitoxin which has removed the fear of death from diphtheria. We may reasonably hope that tuberculosis will before long be in like manner shorn of its terrors; and hence, while we do our best to contend against its present prevalence, it is unnecessary and inexpedient to rear costly edifices for its reception.

